# Glycine Insufficiency as a Metabolic Vulnerability in MASLD: Implications for Precision Nutrition

**DOI:** 10.3390/cimb48101073

**Published:** 2026-10-17

**Authors:** Lixia Wang, Wenxia Xu, Chao Zhang

**Affiliations:** 1 Department of Infectious Disease, Affiliated Jinhua Hospital, Zhejiang University School of Medicine, Jinhua 321000, China; 15067067322@163.com; 2 Jinhua Key Laboratory of Cancer Nutrition and Metabolism Research, Affiliated Jinhua Hospital, Zhejiang University School of Medicine, Jinhua 321000, China; 3 Central Laboratory, Precision Medicine Center, Affiliated Jinhua Hospital, Zhejiang University School of Medicine, Jinhua 321000, China

**Keywords:** glycine, metabolic dysfunction-associated steatotic liver disease, precision nutrition, metabolic vulnerability, one-carbon metabolism, oxidative stress, gut–liver axis

## Abstract

Metabolic dysfunction-associated steatotic liver disease (MASLD) is the most common chronic liver disease worldwide, but effective nutritional interventions remain limited. Low glycine availability is among the most reproducible metabolomic findings in MASLD, and lower glycine levels correlate with greater steatosis, inflammatory activity, and fibrosis severity. This review examines the evidence linking glycine metabolism to MASLD and discusses whether glycine insufficiency may contribute to disease progression beyond its role as a metabolic biomarker. Proposed mechanisms linking altered glycine metabolism to MASLD include changes in SHMT2-related metabolic flux and glycine utilization for glutathione synthesis under oxidative stress. Altered glycine metabolism may be linked to disturbances in one-carbon metabolism, inflammatory signaling, gut–liver axis function, and fibrotic signaling. However, evidence regarding the causal relationships among these processes and the contribution of dietary glycine supply remains limited. Preclinical studies suggest that glycine or glycine-based compounds restore metabolic balance and reduce liver injury, but human trial data remain scarce. Glycine metabolism is therefore a candidate target for precision nutrition. Randomized controlled trials are still needed to determine efficacy, optimal dosing, and which patients are most likely to benefit.

## 1. Introduction

Metabolic dysfunction-associated steatotic liver disease (MASLD) is now the most common chronic liver disease worldwide. It affects roughly 30% of adults, and its prevalence has risen alongside the obesity and type 2 diabetes epidemics [1]. The disease spans a wide spectrum, from simple hepatic steatosis to metabolic dysfunction-associated steatohepatitis (MASH), which is marked by hepatocellular injury, inflammation, and progressive fibrosis, and can ultimately lead to cirrhosis and hepatocellular carcinoma [2]. Although our understanding of its pathogenesis has improved considerably, pharmacological options remain limited. Lifestyle modification with nutritional support is still the mainstay of clinical management [3,4].

The pathogenesis of MASLD is multifactorial, reflecting interactions among genetic susceptibility, environmental triggers, and metabolic dysregulation [1,5]. As the disease progresses, oxidative stress and mitochondrial dysfunction appear early, followed by chronic inflammation and disruption of the gut–liver axis; together, these changes contribute to hepatocellular injury and fibrosis [5,6,7]. Recent metabolomic studies have mapped the metabolic disturbances in MASLD, revealing widespread alterations in amino acid, lipid, and carbohydrate metabolism [8]. One of the most consistent findings across independent cohorts and diverse populations is a significant reduction in circulating and hepatic glycine levels [9,10].

Glycine decline is not limited to a single disease stage. Lower glycine levels correlate with hepatic fat content, insulin resistance, inflammatory activity, and fibrosis severity, suggesting that glycine insufficiency tracks with disease progression [11,12]. Whether this decline is merely a consequence of liver disease or also contributes to pathogenesis remains unclear. Evidence from metabolomic, genetic, and preclinical studies points to the latter possibility, but direct clinical intervention data are still limited. The mechanisms discussed below should therefore be considered hypothesis-driven rather than established. If confirmed, glycine insufficiency could represent a metabolic vulnerability that contributes to the transition from simple steatosis to MASH and fibrosis [13,14].

This review examines glycine’s metabolic roles in hepatic homeostasis and evaluates the clinical and experimental evidence linking glycine insufficiency to MASLD. We discuss the mechanisms that may deplete glycine, the interconnected pathways through which such depletion may contribute to disease progression, and the dietary sources and barriers to clinical translation. Particular attention is given to the potential of glycine-based precision nutrition. Rather than a simple biomarker, glycine insufficiency may represent a modifiable metabolic vulnerability, a concept that could inform nutritional intervention research in MASLD and support individualized care.

## 2. Glycine Metabolism and Hepatic Homeostasis

Glycine is one of the most abundant non-essential amino acids in the human body. Beyond its role in protein synthesis, glycine serves as a precursor for glutathione (GSH), creatine, purines, heme, and bile acids. It also helps maintain redox homeostasis, supports energy metabolism, and sustains cellular function [15,16].

Glycine homeostasis is maintained through a balance between dietary intake and endogenous synthesis [17]. Among these sources, the conversion of serine to glycine catalyzed by serine hydroxymethyltransferase (SHMT) represents the major endogenous pathway. This reaction also generates 5,10-methylenetetrahydrofolate (5,10-CH_2_-THF), a key one-carbon donor that links glycine metabolism to the folate and methionine cycles. Through these interconnected pathways, glycine provides essential substrates for nucleotide biosynthesis, methylation reactions, and cell proliferation [18,19].

The liver is a central organ in glycine metabolism. It not only takes up and releases glycine, but also orchestrates its utilization through several key enzymes. Among these, glycine N-methyltransferase (GNMT) plays a particularly important role in maintaining methylation homeostasis by regulating intracellular S-adenosylmethionine (SAM) levels [17,20]. GNMT catalyzes the transfer of a methyl group from SAM to glycine, generating sarcosine (monomethylglycine) and S-adenosylhomocysteine (SAH). This reaction serves as a critical sink for excess SAM, preventing its toxic accumulation [17,20]. Accordingly, GNMT deficiency leads to abnormal accumulation of SAM and is accompanied by marked hepatic steatosis, inflammation, and liver injury [21]. Conversely, the glycine cleavage system (GCS) provides an alternative route for glycine catabolism, generating 5,10-CH_2_-THF and ammonia, thereby contributing to one-carbon pool maintenance [17,22].

Thus, glycine sits at the intersection of multiple metabolic networks that are essential for hepatic function [20]. It is not surprising, therefore, that disruptions in glycine metabolism have been implicated in the pathogenesis of metabolic liver diseases. As we will discuss in the following sections, the emerging evidence points to glycine insufficiency as a consistent feature of MASLD, with far-reaching consequences for disease progression [17].

## 3. Glycine Insufficiency in MASLD: Evidence and Underlying Mechanisms

### 3.1. Clinical Evidence for Reduced Glycine Levels in Patients with MASLD

Metabolomic technologies have enabled the systematic mapping of metabolic disturbances in MASLD. Across studies using plasma, serum, and liver tissue, the most consistent finding is a significant reduction in glycine levels [4,9,23]. This reduction has been observed across diverse ethnic groups, geographic regions, and analytical platforms, underscoring its robustness as a metabolic signature of the disease [24,25].

Reduced circulating glycine levels correlate with several key clinical parameters. Studies have shown that lower glycine is associated with higher hepatic fat content, more severe insulin resistance, and elevated inflammatory markers [11,12]. Importantly, as the disease progresses from simple steatosis to MASH and fibrosis, glycine levels decline further, suggesting a relationship between glycine depletion and disease severity [4]. In a large cross-sectional study, Hasegawa et al. demonstrated that serum glycine levels were significantly lower in patients with NAFLD and that this reduction was independent of insulin resistance, pointing to a direct link between glycine metabolism and liver pathology [9]. Similarly, Zhou et al. identified low glycine as a noninvasive marker for detecting NASH, with diagnostic performance comparable to more complex biomarker panels [10].

The clinical significance of these findings extends beyond diagnosis. Low glycine levels have been proposed as a risk marker for MASLD and its complications [23,26]. A small clinical trial reported that dietary glycine supplementation improved markers of MASLD severity in individuals with severe obesity [27]. This finding, though preliminary, is consistent with the possibility that glycine insufficiency may not merely be a marker, but could contribute to disease progression. However, the study was limited by its small sample size and non-randomized design, and these results require confirmation in adequately powered randomized controlled trials. Taken together, these clinical data indicate that glycine insufficiency is a reproducible and clinically relevant feature of MASLD, and raise the question of whether it contributes to disease pathogenesis.

### 3.2. Mechanisms Underlying Glycine Insufficiency

The mechanisms driving glycine depletion in MASLD are multifaceted, involving metabolic reprogramming, enhanced oxidative stress, and impaired supply.

#### 3.2.1. Metabolic Reprogramming

In the setting of lipid overload, hepatocytes upregulate mitochondrial serine hydroxymethyltransferase 2 (SHMT2) as part of a compensatory metabolic adaptation [13]. This enzyme catalyzes the reversible interconversion of serine and glycine. Under normal conditions, the forward reaction converts serine to glycine and generates 5,10-methylenetetrahydrofolate (5,10-CH_2_-THF), a one-carbon donor [28]. Under conditions of metabolic stress, however, the reverse reaction—the conversion of glycine to serine—can become dominant, consuming glycine and diverting it away from other glycine-dependent reactions [16]. Ghrayeb et al. reported that this reverse SHMT2 activity contributes to glycine depletion in MASLD, suggesting a mechanistic link between metabolic stress and reduced glycine availability [13]. However, this mechanism has been described in only one study and awaits independent validation.

#### 3.2.2. Oxidative Stress and Increased Demand

As MASLD progresses, oxidative stress intensifies, driving excessive production of reactive oxygen species (ROS) [29]. GSH is a major antioxidant that neutralizes ROS, and its consumption under oxidative stress increases the demand for glycine as a GSH substrate [30]. When supply cannot meet this demand, functional glycine insufficiency develops [31].

#### 3.2.3. Impaired Endogenous Synthesis

MASLD is often accompanied by mitochondrial dysfunction and generalized disruption of amino acid metabolism, both of which can compromise the liver’s capacity for endogenous glycine synthesis [32,33,34]. Mitochondrial dysfunction, in particular, may impair the glycine cleavage system, which normally contributes to one-carbon metabolism, and may also affect other glycine-generating pathways, further limiting glycine availability [34].

This framework may explain the progressive decline in glycine levels observed across clinical and experimental studies and suggests that glycine insufficiency is a functionally important feature of MASLD rather than a passive bystander. The four mechanisms underlying glycine insufficiency are summarized in Figure 1.

### 3.3. Glycine and Interconnected Metabolic Pathways

Glycine does not act alone. In MASLD, glycine insufficiency occurs alongside changes in several related metabolic pathways, and changes in one pathway may affect others.

#### 3.3.1. The Serine–Glycine Axis

Glycine and serine are interconverted by SHMT1 and SHMT2, and this axis is altered in MASLD [35]. Under lipid overload, increased reverse SHMT2 flux has been proposed to convert glycine to serine, contributing to glycine depletion [13]. Because this reaction consumes 5,10-methylenetetrahydrofolate, altered SHMT2 activity may also influence the distribution of folate-bound one-carbon intermediates [36,37]. However, the net effects on one-carbon metabolism depend on the coordinated activity of SHMT enzymes, the glycine cleavage system, and other folate-dependent reactions [38].

#### 3.3.2. The Methionine Cycle

Glycine is the methyl acceptor in the GNMT reaction, which converts SAM to SAH and sarcosine [39]. When glycine is limiting, GNMT flux may decline, reducing SAM catabolism and potentially altering the SAM/SAH ratio. However, the net effect on methylation homeostasis depends on the coordinated activity of the methionine cycle, the folate cycle, and other methyl donor pathways [20,39]. GNMT deficiency itself leads to SAM accumulation accompanied by steatosis, inflammation, and liver injury, indicating that intact glycine-dependent SAM catabolism is required for hepatic metabolic homeostasis [40,41].

#### 3.3.3. The Transsulfuration Pathway

The methionine cycle is linked to glutathione synthesis through the transsulfuration pathway, which converts homocysteine to cysteine, a key substrate for GSH synthesis [42,43]. Glycine and cysteine are both required for GSH synthesis, and when glycine is limiting, the coordinated supply of substrates for GSH synthesis is impaired [44,45]. This pathway connects glycine metabolism to the methionine cycle and to antioxidant defense.

#### 3.3.4. BCAA Metabolism

Branched-chain amino acids (BCAAs) are consistently elevated in MASLD and are inversely associated with glycine levels [46,47]. Low serum glycine has been shown to strengthen the association between BCAAs and impaired insulin sensitivity, suggesting that the BCAA–glycine relationship may contribute to metabolic dysfunction [46]. The mechanisms linking BCAA elevation to glycine depletion remain incompletely defined, although shared nitrogen trafficking and one-carbon metabolism have been proposed [48,49].

Overall, these observations suggest that glycine insufficiency is one component of a broader set of related metabolic changes rather than an isolated metabolic defect, and that the pathological significance of low glycine may be better understood in this context.

### 3.4. Glycine Insufficiency: Cause or Consequence?

Whether glycine insufficiency is a cause or a consequence of MASLD remains debated, and the available evidence is still limited.

Early in the disease course, glycine depletion appears to be largely a consequence of metabolic adaptation to lipid overload. To maintain metabolic homeostasis, hepatocytes upregulate one-carbon activity and shunt glycine toward serine synthesis, gradually lowering glycine levels in the liver and circulation [13,38]. From this perspective, glycine depletion is initially a metabolic consequence of disease-associated stress. The transition from consequence to contributor may depend on whether glycine depletion reaches a threshold that impairs glutathione synthesis and methylation homeostasis [13].

However, when glycine insufficiency persists, its biological effects become increasingly consequential. Lower glycine levels track with higher MASLD risk and greater disease severity, and Mendelian randomization analyses suggest a causal contribution [9,25,50]. In animal models, glycine supplementation mitigates disease progression, further supporting the idea that glycine insufficiency is not merely a marker, but may also contribute to disease progression [51,52].

Overall, these observations suggest that glycine insufficiency may act as a metabolic amplifier in MASLD. It may initially arise as a consequence of disease-associated metabolic alterations, but when it persists, it may in turn exacerbate oxidative stress and impair antioxidant defense [13]. This has been proposed to create a self-reinforcing cycle, although direct evidence for such a feedback loop in MASLD is still lacking. This framework remains a hypothesis that requires validation in prospective clinical studies. If confirmed, it would shift the view of glycine from a metabolic biomarker to a potential therapeutic target.

## 4. Mechanisms by Which Glycine Insufficiency May Contribute to MASLD Progression

Glycine insufficiency may promote MASLD progression through multiple interconnected biological pathways. As a key metabolic node linking redox homeostasis, one-carbon metabolism, immune regulation, and tissue remodeling, glycine insufficiency has the potential to influence hepatic pathophysiology at multiple levels [17].

### 4.1. The γ-Glutamyl Cycle and Glutathione Synthesis: The Core Redox Node

Glycine is an essential substrate for glutathione (GSH) synthesis. In the terminal step of the γ-glutamyl cycle, glutathione synthetase catalyzes the conjugation of γ-glutamylcysteine with glycine to form GSH. Because hepatic GSH turnover is high and hepatocytes cannot store GSH in large excess, a decline in glycine availability may limit the cell’s antioxidant capacity, particularly when glycine becomes limiting relative to demand [44,53].

In MASLD, lipid overload further increases GSH demand, and when this demand exceeds supply, GSH synthesis may decline and ROS scavenging may be impaired [54]. The resulting oxidative stress promotes lipid peroxidation and hepatocyte injury, and it also further increases GSH demand, creating a self-reinforcing cycle between glycine depletion and redox imbalance [55]. This mechanism is supported by preclinical studies in which glycine or glycine-based treatment restored GSH levels and limited liver injury. Rom et al. showed that glycine-based treatment ameliorates NAFLD in mice by enhancing glutathione synthesis, and DT-109, a glycine-based tripeptide, similarly increased GSH and attenuated hepatic steatosis and inflammation in animal models [14,56]. Thus, impaired GSH synthesis may result from glycine insufficiency and may contribute to MASLD progression. This redox node may be upstream of the inflammatory, gut–liver axis, and fibrogenic pathways discussed below, as GSH depletion can increase oxidative stress, which in turn may contribute to inflammatory signaling and fibrogenesis [55].

### 4.2. One-Carbon Metabolism: SHMT2 Reverse Flux, Folate Cycle, and SAM/SAH-Dependent Epigenetic Reprogramming

Reverse SHMT2 flux has been proposed to convert glycine to serine under lipid overload, which may consume glycine and reduce the hepatic glycine pool. This mechanism has been described in only one study and awaits independent validation. This metabolic shift may reduce glycine availability for other biosynthetic demands, including glutathione synthesis, particularly when glycine becomes limiting relative to cellular demand [16]. In addition, glycine insufficiency may perturb methylation homeostasis through alterations in GNMT activity and one-carbon metabolism, potentially influencing epigenetic regulation [57]. Dysregulated N6-methyladenosine (m6A) RNA methylation has been implicated in lipid accumulation and inflammatory responses in experimental MASLD models [58,59]. However, whether glycine insufficiency directly modulates m6A patterns through changes in GNMT activity, SAM/SAH balance, or other metabolic mechanisms remains unclear and requires targeted investigation.

### 4.3. Anti-Inflammatory Signaling and Gut–Liver Axis Dysfunction

#### 4.3.1. GlyR-Mediated Anti-Inflammatory Signaling and NF-κB Suppression

Chronic low-grade inflammation is a major driving force in MASLD progression [60]. Glycine exerts anti-inflammatory effects through multiple mechanisms [61]. In Kupffer cells, glycine acts through the glycine receptor (GlyR), a glycine-gated chloride channel whose expression has been confirmed in these cells [62]. GlyR activation suppresses NF-κB signaling, thereby reducing the release of pro-inflammatory cytokines such as TNF-α, IL-1β, and IL-6. This glycinergic signaling suppresses hepatic inflammatory activation [63,64,65,66]. A decline in glycine availability weakens this endogenous anti-inflammatory capacity, facilitating the persistence of chronic hepatic inflammation [61].

Glycine insufficiency also affects redox balance through this pathway [13]. Inflammatory signaling increases ROS production and raises glutathione demand, which further consumes glycine and aggravates its insufficiency [11]. Conversely, glycine depletion impairs glutathione synthesis and weakens antioxidant defense, allowing oxidative stress to sustain inflammatory activation [67]. Thus, glycine insufficiency, redox imbalance, and chronic inflammation may reinforce each other during MASLD progression [14].

#### 4.3.2. Gut–Liver Axis: Intestinal Barrier, LPS/TLR4 Signaling, and Microbial Glycine Metabolism

Glycine also contributes to gut microbial homeostasis. Preclinical evidence indicates that glycine supplementation improves gut microbial composition, strengthens the intestinal barrier, and reduces endotoxin exposure [68,69,70]. Preclinical studies have shown that glycine supplementation improves intestinal barrier integrity and protects against LPS-induced intestinal injury [71]. Increased intestinal permeability and translocation of bacterial products such as LPS have been linked to MASLD-associated hepatic inflammation [72]. However, direct evidence that glycine insufficiency increases intestinal permeability and promotes hepatic inflammation in MASLD is still limited. Hepatic LPS then activates TLR4 on Kupffer cells, triggering NF-κB nuclear translocation and NLRP3 inflammasome assembly [73]. This in turn promotes the release of pro-inflammatory cytokines including IL-1β and TNF-α [74]. These findings are derived mainly from preclinical models, and clinical evidence in MASLD patients remains limited [51,52]. Nonetheless, they suggest that gut–liver axis disruption may link glycine insufficiency to inflammatory progression in MASLD.

### 4.4. TGF-β1/SMAD Signaling and HSC Activation in Fibrogenesis

Liver fibrosis marks a critical stage in MASLD progression toward end-stage liver disease, with hepatic stellate cell (HSC) activation and excessive extracellular matrix (ECM) deposition as its hallmarks [75].

Glycine may influence fibrogenesis through its effects on inflammation and oxidative stress, both of which promote HSC activation [76]. In CCl_4_-induced fibrosis models, dietary glycine supplementation attenuated liver fibrosis by suppressing Kupffer cell activation, reducing TGF-β1 and α-SMA expression, and limiting collagen deposition [77]. CCl_4_-induced fibrosis is not, however, a MASLD model, and extrapolation of these findings to MASLD-associated fibrosis requires caution. In MASLD animal models, DT-109—a glycine-based tripeptide (Gly-Gly-Leu)—reduced hepatic steatosis and inflammation and also attenuated fibrosis, potentially through enhanced glutathione synthesis, increased fatty acid oxidation, and restored metabolic homeostasis [14,56].

Recent work on metabolic reprogramming in liver fibrosis has provided further insight into the potential mechanisms. Activated HSCs undergo profound metabolic reprogramming to meet the energetic demands of continuous ECM synthesis, and the one-carbon network—including the serine–glycine pathway—may participate in this process [76]. Meliton et al. demonstrated that TGF-β induces the expression of mitochondrial one-carbon pathway enzymes, including MTHFD2, and that MTHFD2 is required for TGF-β-induced glycine accumulation and collagen production [78]. Pharmacological inhibition of MTHFD2 ameliorated fibrotic responses in vivo, suggesting that mitochondrial one-carbon metabolism contributes to fibrosis. Notably, SHMT2 may function differently at different stages of MASLD: hepatic SHMT2 deficiency reduces steatosis but exacerbates inflammation and fibrosis in mice, suggesting that the relationship between glycine metabolism and fibrosis is not straightforward [38]. Direct evidence for this mechanism in MASLD-associated fibrosis is still limited, and its connection to glycine supplementation requires further investigation.

Overall, these findings suggest that glycine insufficiency may promote MASLD-associated fibrosis through impaired one-carbon metabolism and reduced antioxidant defense, although direct evidence in MASLD remains limited. Future work should combine systems-level metabolic profiling with mechanistic studies to clarify how glycine metabolism operates at different stages of liver fibrosis and to inform precision nutritional strategies.

## 5. Dietary Sources of Glycine and Nutritional Exposure

Glycine is derived from two major sources in humans: endogenous synthesis and dietary intake. Under physiological conditions, SHMT-mediated conversion of serine to glycine supplies most of the body’s glycine needs. However, under metabolic conditions such as obesity, insulin resistance, and MASLD, endogenous glycine synthesis is reduced while glycine utilization is increased, rendering glycine a conditionally essential amino acid [17,49,79].

Diet is an important source of glycine, and glycine content varies widely among foods. According to USDA FoodData Central, some animal-derived foods are rich in glycine, but certain plant-derived foods—including defatted soy flour, peanut butter, almond butter, almonds, and flaxseed—also contain considerable amounts (Table 1). In animal-derived foods, high glycine content largely reflects collagen, which contains glycine at approximately one-third of its amino acid residues; connective tissues, skin, bones, and tendons are therefore major animal-derived sources. Plant-derived foods can also contribute to dietary glycine intake despite lacking collagen.

Dietary intake does not directly determine circulating glycine levels. In 392 men from the EPIC-Oxford cohort, meat eaters had the highest intake of all 18 amino acids and vegans the lowest, with a difference of up to 47% [80]. Plasma glycine showed the opposite pattern: vegans had the highest levels despite the lowest intake, whereas meat eaters had the lowest despite the highest intake [80]. Circulating glycine therefore depends on endogenous synthesis, intestinal and microbial utilization, tissue uptake, and metabolic reprogramming, not on dietary intake alone [17].

Collagen is a major contributor to glycine intake from animal-derived foods, but not the only dietary source. The Irish National Adult Nutrition Survey estimated average dietary collagen intake in European adults at roughly 3 g/day—about 4% of daily protein intake and well below the 15–30 g/day used in intervention studies to promote collagen synthesis [81]. Low collagen intake may thus indicate limited glycine supply from animal sources in typical Western diets, but it does not necessarily indicate systemic glycine insufficiency, because plant-derived foods also contribute. Dietary supply is only one determinant of circulating glycine [28,81,82].

These observations have important implications for MASLD. Patients with metabolic dysfunction may have increased glycine requirements due to elevated oxidative stress and inflammation [83]. Although dietary intake may contribute, endogenous synthesis and consumption are likely to play a larger role in determining glycine availability in MASLD [14]. In particular, enhanced reverse SHMT2 flux and impaired endogenous glycine synthesis may be more important than dietary intake in contributing to glycine insufficiency in MASLD [38].

## 6. Precision Nutrition and Clinical Translation of Glycine

### 6.1. From Metabolic Biomarker to Nutritional Intervention Target

MASLD is a metabolically heterogeneous disease. Interindividual differences in amino acid metabolism, lipid metabolism, mitochondrial function, and inflammatory status indicate that a uniform nutritional intervention is unlikely to be suitable for all patients [11,84]. Glycine, however, occupies a unique position: it reflects metabolic disturbances in MASLD and is proposed to regulate several key metabolic networks, making it both a biomarker and a potential nutritional target [4]. It should be emphasized, however, that glycine-based precision nutrition remains a hypothesis-driven strategy [14]. No clinically validated glycine intervention for MASLD currently exists, and the strategies discussed in this section should be regarded as hypothesis-generating rather than clinically established.

Precision medicine approaches propose integrating metabolomic profiles, genetic information, and clinical phenotypes to stratify patients, allowing targeted interventions for those most likely to respond [85]. In the context of glycine, this might involve identifying patients with the lowest baseline glycine levels, those with genetic variants affecting glycine metabolism (e.g., SHMT2 or GNMT polymorphisms), or those with specific gut microbial compositions that influence glycine bioavailability. By targeting glycine supplementation to these subgroups, it may be possible to improve efficacy and avoid unnecessary interventions in those unlikely to benefit [86].

At present, these proposed stratification criteria—low baseline glycine levels, SHMT2 or GNMT polymorphisms, and gut microbial composition—remain experimental. No clinically validated biomarker of glycine treatment response currently exists for MASLD [25,27].

### 6.2. Challenges in Clinical Translation

Despite the promising experimental evidence, substantial challenges remain before glycine-based interventions can be translated into routine clinical practice [87,88,89].

#### 6.2.1. Limited Human Trial Data

Most evidence linking glycine metabolism to MASLD comes from cell-based and animal studies; rigorous clinical trials of glycine supplementation in MASLD patients are scarce [87]. A recent systematic review by Soh et al. identified only a limited number of human studies on glycine administration, with considerable heterogeneity in study populations, supplementation doses, intervention durations, and outcome measures, making direct comparisons difficult and limiting the translation of current evidence into clinical practice [87].

#### 6.2.2. Disease Heterogeneity

MASLD encompasses a broad spectrum of metabolic phenotypes. Obesity, type 2 diabetes, genetic background, and gut microbial composition may all influence metabolic characteristics and responsiveness to nutritional interventions [90,91]. Patients with different metabolic phenotypes are likely to exhibit distinct responses to glycine-based interventions. Therefore, future nutritional strategies should move beyond a “one-size-fits-all” approach toward precision supplementation tailored to individual metabolic characteristics.

#### 6.2.3. Optimal Dosing and Safety

The optimal dose, duration, and formulation of glycine supplementation for MASLD have not been established. Most preclinical studies used doses that may not be directly translatable to humans, and the long-term safety of high-dose glycine supplementation remains unknown [27,92]. Moreover, potential interactions between glycine supplementation and medications commonly used in MASLD patients remain insufficiently studied.

#### 6.2.4. Patient Adherence and Practical Implementation

Even if efficacy is established, implementing glycine-based nutritional interventions in clinical practice will require addressing practical barriers, including patient adherence to dietary modifications, the availability and cost of supplements, and the integration of nutritional interventions into routine hepatology care [93,94].

## 7. Discussion

MASLD progression involves multiple pathogenic mechanisms, including lipid dysregulation, oxidative stress, chronic inflammation, one-carbon imbalance, and gut–liver axis disruption [7]. Metabolomic, genetic, and experimental studies point to reduced glycine availability as a reproducible metabolic feature of MASLD [13]. Importantly, glycine depletion correlates with steatosis, inflammation, and fibrosis severity, and preclinical evidence suggests that it may actively contribute to disease progression [14]. However, this remains a hypothesis-driven interpretation, as direct clinical intervention data are still lacking.

If glycine insufficiency reflects a state of metabolic vulnerability, several implications for MASLD pathogenesis follow. Glycine metabolism may provide an entry point for nutritional intervention that complements existing lifestyle and pharmacological approaches [14]. This perspective also calls for greater consideration of nutrient interactions and individual metabolic phenotypes in therapeutic design [11]. More broadly, it supports the view that MASLD is better understood as a disease of metabolic dysregulation rather than simply a disorder of fat storage [95].

However, several questions remain. Mechanistically, it remains unclear what initiates the metabolic reprogramming that contributes to glycine depletion, or whether the consequences of altered glycine metabolism differ across disease stages. As discussed in Section 4.4, the effects of SHMT2 may not be uniform across MASLD progression [38]. Addressing these gaps will be necessary before glycine-based interventions can be considered for clinical use. A further challenge is that glycine insufficiency lacks specific clinical symptoms, which is why it has been described as a “silent” metabolic vulnerability and why biochemical markers, such as plasma glycine levels, are needed for its identification [87]. Key unresolved clinical issues include optimal dosing, bioavailability, formulation, long-term safety, and potential drug interactions, as discussed in Section 6.2. The causes of glycine insufficiency are also multifactorial, including increased consumption, impaired synthesis, and inadequate dietary intake.

Despite these gaps, glycine insufficiency may still be useful as an identifiable and potentially modifiable state of metabolic vulnerability that can inform nutritional intervention research in MASLD [86]. Rather than recommending increased protein or amino acid intake for all patients, future work should identify who is most likely to benefit from glycine-based strategies and how these strategies can be integrated with other therapeutic approaches [85]. Adequately powered randomized controlled trials are needed to assess the effects of glycine supplementation on histological endpoints, metabolic outcomes, and clinical prognosis [96]. Metabolomics, genetics, and artificial intelligence-assisted analyses may help stratify patients [97]. Glycine should also be considered within the broader metabolic network, including its interactions with diet, lifestyle, and other nutritional factors [98]. Clinical translation will further require educating healthcare providers and integrating nutritional interventions into routine hepatology care [93]. The interconnected pathways through which glycine insufficiency may contribute to steatosis, inflammation, and fibrosis, as well as the proposed precision nutrition strategies, are summarized in Figure 2.

## 8. Conclusions

Glycine insufficiency is a consistent feature of MASLD that tracks with disease severity and correlates with oxidative stress, one-carbon disruption, inflammation, and fibrosis. The evidence reviewed here suggests that glycine insufficiency is a reproducible biomarker and may also reflect a metabolically vulnerable state that can contribute to disease progression. In animal models, glycine or glycine-based compounds restore metabolic balance and reduce liver injury, but human data remain sparse. Randomized controlled trials are needed to define the efficacy, optimal dosing, and safety of glycine-based interventions in patients with MASLD. Mechanistic studies should be integrated with omics approaches and clinical phenotyping to identify patients most likely to benefit. Generalized supplementation is unlikely to benefit all patients; precision nutrition guided by metabolic traits may be more promising. If glycine insufficiency is a modifiable vulnerability, targeting glycine metabolism may become a practical strategy for individualized management of MASLD. At present, however, this remains a hypothesis that requires validation in randomized controlled trials.

## Figures and Tables

**Figure 1:**
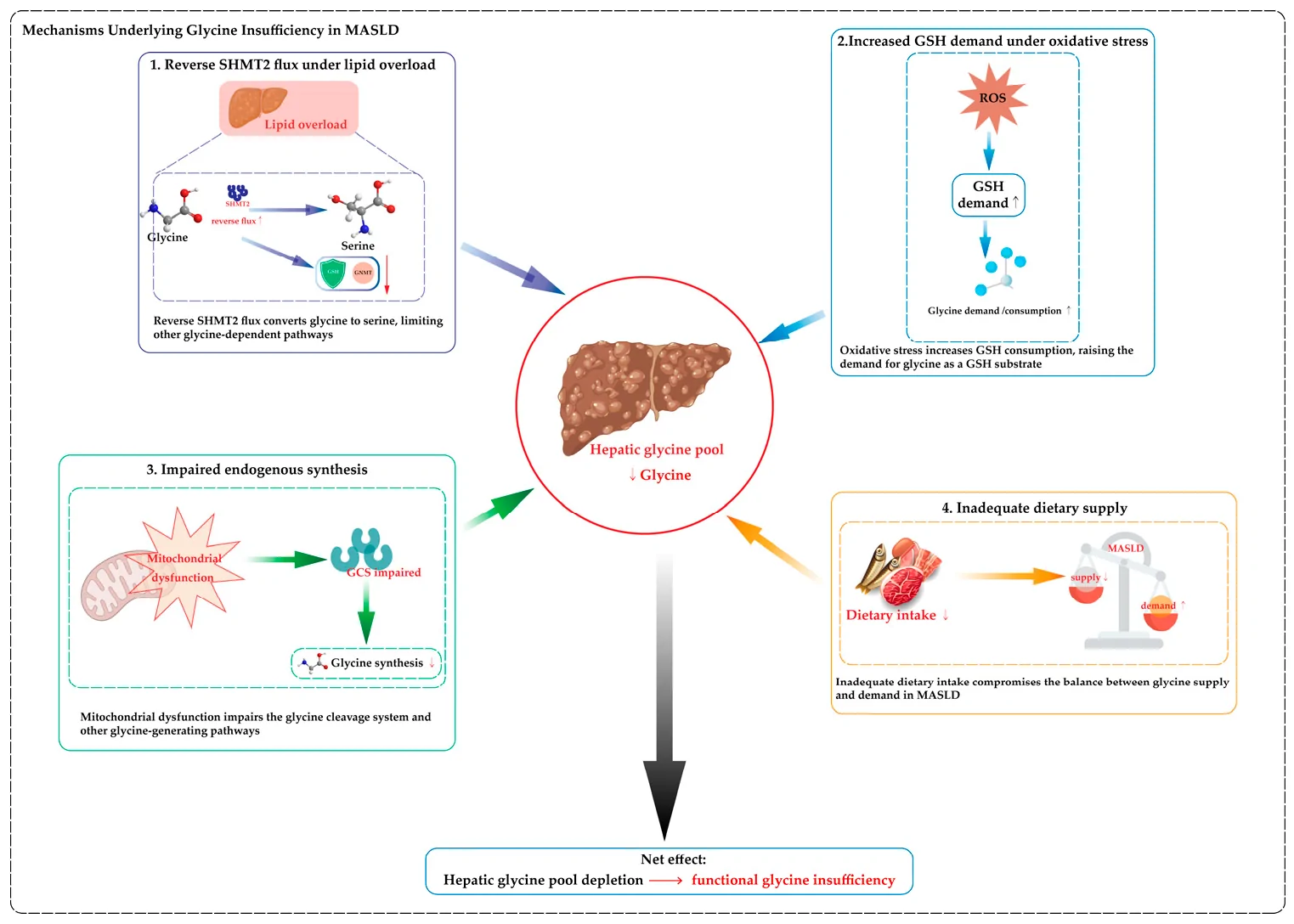
Mechanisms underlying glycine insufficiency in MASLD. Four mechanisms contribute to hepatic glycine pool depletion.Upward arrows (↑) indicate an increase or upregulation, and downward arrows (↓) indicate a decrease or downregulation.

**Figure 2:**
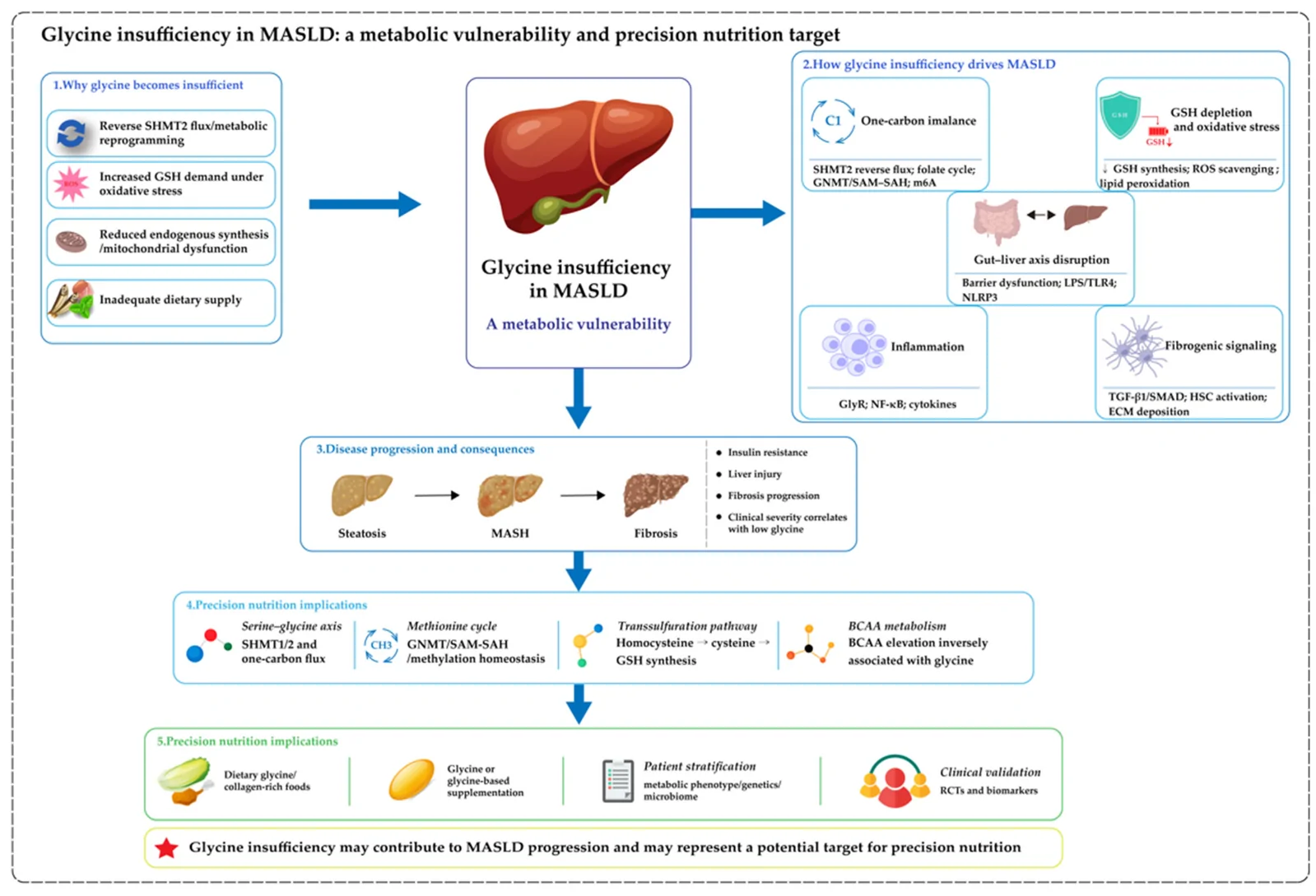
Schematic overview of glycine insufficiency in MASLD pathogenesis and precision nutrition implications.Upward arrows (↑) indicate an increase or upregulation, and downward arrows (↓) indicate a decrease or downregulation.The red star indicates emphasis.

**Table 1:** Glycine content in representative foods (data adapted from USDA FoodData Central, https://fdc.nal.usda.gov/food-search?component=1225 (accessed on 15 August 2026)).

Food Category	Representative Food	Glycine (g/100 g Edible Portion)	FDC ID
Nuts	Almonds, raw	1.430	2346393
	Pine nuts, raw	0.730	2346392
	Pecans, raw	0.492	2346395
	Walnuts, raw	0.690	2346394
Seeds	Ground flaxseed	1.390	2262075
Nut and seed butters	Tahini (sesame butter)	1.220	2262073
	Peanut butter	1.690	2262072
	Almond butter	1.650	2262074
Soy products	Defatted soy flour	1.790	1104705
	Full-fat soy flour	1.350	1104766
Eggs	Whole egg	0.408	748967
Dairy products	Whole milk (3.25% fat)	0.069	746782
	Reduced-fat milk (2% fat)	0.068	746778
	Skim milk	0.071	746776
Fish	Alaska pollock, raw	0.705	2768188
Meat	Beef	0.940	746779

Note. Glycine content is expressed per 100 g of edible portion. FDC ID, FoodData Central identification number. These values illustrate differences in dietary glycine content across representative food categories and are not direct estimates of circulating glycine availability. The database values do not consider cooking methods, food matrix effects, digestibility, intestinal and microbial utilization, or individual metabolic differences. Dietary glycine intake is only one factor influencing circulating glycine levels, as discussed in Section 5.

## Data Availability

No new data were created or analyzed in this study. Data sharing is not applicable to this article.

## Abbreviations

ECM	extracellular matrix
GCS	glycine cleavage system
GNMT	glycine N-methyltransferase
GSH	glutathione
HSC	hepatic stellate cell
LPS	lipopolysaccharide
MASLD	metabolic dysfunction-associated steatotic liver disease
MTHFR	methylenetetrahydrofolate reductase
m^6^A	N^6^-methyladenosine
ROS	reactive oxygen species
SAM	S-adenosylmethionine
SAH	S-adenosylhomocysteine
SHMT	serine hydroxymethyltransferase
SHMT2	mitochondrial serine hydroxymethyltransferase
TGF-β	transforming growth factor-β
TLR4	Toll-like receptor 4
α-SMA	α-smooth muscle actin
